# Physiological Slowing and Upregulation of Inhibition in Cortex Are Correlated with Behavioral Deficits in Protein Malnourished Rats

**DOI:** 10.1371/journal.pone.0076556

**Published:** 2013-10-03

**Authors:** Rahul Chaudhary, Manisha Chugh, Ziauddin Darokhan, Raghu Ram Katreddi, Renuka Ramachandra, V. Rema

**Affiliations:** National Brain Research Centre, Manesar, Haryana, India; Hôpital Robert Debré, France

## Abstract

Protein malnutrition during early development has been correlated with cognitive and learning disabilities in children, but the neuronal deficits caused by long-term protein deficiency are not well understood. We exposed rats from gestation up to adulthood to a protein-deficient (PD) diet, to emulate chronic protein malnutrition in humans. The offspring exhibited significantly impaired performance on the ‘Gap-crossing’ (GC) task after reaching maturity, a behavior that has been shown to depend on normal functioning of the somatosensory cortex. The physiological state of the somatosensory cortex was examined to determine neuronal correlates of the deficits in behavior. Extracellular multi-unit recording from layer 4 (L4) neurons that receive direct thalamocortical inputs and layers 2/3 (L2/3) neurons that are dominated by intracortical connections in the whisker-barrel cortex of PD rats exhibited significantly low spontaneous activity and depressed responses to whisker stimulation. L4 neurons were more severely affected than L2/3 neurons. The response onset was significantly delayed in L4 cells. The peak response latency of L4 and L2/3 neurons was delayed significantly. In L2/3 and L4 of the barrel cortex there was a substantial increase in GAD65 (112% over controls) and much smaller increase in NMDAR1 (12-20%), suggesting enhanced inhibition in the PD cortex. These results show that chronic protein deficiency negatively affects both thalamo-cortical and cortico-cortical transmission during somatosensory information processing. The findings support the interpretation that sustained protein deficiency interferes with features of cortical sensory processing that are likely to underlie the cognitive impairments reported in humans who have suffered from prolonged protein deficiency.

## Introduction

Protein deficiency in-utero and during early childhood has been correlated with detrimental effects on cognitive function [1-5] and on brain structures such as cerebral atrophy ventricular dilatation [6,7], myelination delay [8] and abnormal dendritic spines [9,10]. Reports of abnormalities in electroencephalography (EEG) patterns [11,12], auditory brainstem potentials [13,14], auditory [15] and visual [14] evoked potential in children raised on a protein-deficient (PD) diet indicate that consumption of PD diet leads to impairments in neuronal activity and sensory processing.

Similar to humans, animals exposed to PD diet during early development have cognitive and behavioral deficits [3], abnormalities in brain structures [16] including alterations in apical and basilar dendritic arborization and orientation of pyramidal cells in the neocortex [17-19], reduction in dendritic branching, number of spines, synapses and mossy fibre formation in hippocampus [20-23], malformation of hypothalamic nuclei [24] and anatomical changes in somatosensory cortex [25]. Exposure of animals to protein deficiency has also been correlated with electrophysiological dysfunctions such as increase in frequency of miniature postsynaptic currents in CA3 interneurons [26] and in CA1 [27] of the hippocampus, reduction in population excitatory post synaptic potential amplitude in the hippocampus [28-30] as well as alterations in EEG [31] and prepulse inhibition [32]. Field potential recording from neocortex [33], entorhinal cortex [34] and hippocampus [35-38] showed that prenatal protein malnourished rats had impairment in induction and maintenance of long term potentiation.

Given the vulnerability of the developing brain to PD diets, most of the studies using animal models have focused on the effect of early perinatal protein deficiency. However, due to poverty-related socioeconomic conditions in many regions, protein deficiency can be present throughout life. In this study, we tested the hypothesis that chronic exposure to protein deficiency from gestation to adulthood can impair sensory information processing and lead to behavioral deficits. We examined sensory processing in the somatosensory whisker barrel cortex of PD rats to quantify deficits in (i) whisker-dependent somatosensory behavior; (ii) the response properties of cortical neurons that are driven by direct thalamic inputs; (iii) the response of neurons generated by intracortical connections and (iv) levels of excitation and inhibition in the somatosensory cortex. Our results provide evidence that chronic protein deficiency leads to profound deficits in sensory processing and behavior.

## Methods

Out-bred, Long-Evans rats were obtained from the National Brain Research Centre for this study. All experimental procedures used in this study were approved by the Institutional Animal Care and Use Committee of National Brain Research Centre and were in accordance with NIH guidelines. A total of 57 rats (13 adult female dams, 7 adult males and 37 pups) were used for this study. Experiments were designed to minimize the number of animals needed and the discomfort of individual animals during experimental procedures.

### Exposure of rats to protein-deficient and control diets

Female rats (2 months old) were housed in standard laboratory conditions in 12 hr light/dark cycle at 22°C with ad libitum access to water and to one of the three diets: (i) PD diet (7% casein, n = 6) or (ii) control diet (CD, 24% casein, n = 4) or laboratory chow (LC, n = 3). All three diets were isocaloric. The constituents in the PD diet were casein 7.1%, starch 65.1%, sucrose 10%, cellulose 6%, refined groundnut oil 7%, mineral mixture 3.5%, vitamin mixture 1%, L-cystine 0.3%. The control diet composition was casein 24.2%, starch 50.1%, sucrose 8%, cellulose 6%, refined groundnut oil 7%, mineral mixture 3.5%, vitamin mixture 1%, L-cystine 0.3%. Whereas, the laboratory chow was composed of 20-22% crude protein, 5-6% crude fat, 5% crude fibre, 10% moisture and fortified with recommended quantities of minerals and essential vitamins. The protein-deficient diet and the control diet were obtained from National Institute of Nutrition, Hyderabad, India. The dietary paradigm was started three weeks prior to conception and continued throughout gestation and lactation. Following birth each litter was culled to 8 pups. The day of birth was considered as postnatal day (P) 0. The PD group consisted of all pups born to mothers fed protein-deficient diet. The pups born to mothers ingesting control diet constituted the CD group. The LC group comprised of pups born to laboratory chow fed mothers. The pups were weaned on P21 and housed in pairs. They were maintained on the same diet as their mothers until the termination of experiment. Hence, exposure of pups to protein deficiency was through the entire gestation period, during lactation and continued until maturity. The laboratory chow fed group was included in this study as an additional control to account for any deviation that might arise due to consumption of purified diets, i.e. PD and control diets. Food intake and physical appearance of the pups were monitored daily. On attaining adulthood (two month age) rats were randomly selected from each group for conducting behavior, electrophysiology and immunohistochemistry experiments.

### Behavioral testing: ‘Gap-crossing’ task

Adult PD (n =5), CD (n = 5) and LC (n =5) rats were tested on their performance of the Gap-Crossing (GC) task to assess their whisker mediated somatosensory behavior. The GC apparatus consisted of a fixed start-platform and a movable reward-platform [39]. The GC behavior was tested in total darkness to eliminate visual cues. Food was removed 12 hrs prior to behavioral testing. During habituation (2 days) there was no gap between the platforms and the rats (2-3 selected randomly from each litter) explored the GC apparatus to find the food reward (a chocolate flavored cornflake) placed on the reward-platform. The rats were trained to cross gaps with 1cm increments between the platforms to obtain the reward. The gaps between the platforms were increased and decreased randomly to induce contact of the reward-platform by the whiskers prior to crossing. Two rats that responded by freezing and indiscriminate jumping were excluded. Individual trials were terminated after a maximum time of 180 s [40]. The GC ability was tested for the next three days and video recorded, using infrared night vision equipment (Sony Handycam), for offline analysis. Subsequent to testing the GC performance, the weight and length of each rat were determined. Frame by frame analyses of the videos were carried out manually by an investigator, blind to the experimental conditions. Performance on each trial was evaluated for successful and unsuccessful attempts. For each attempt, the width of gap and the time spent by the rat at the edge of the start platform were noted.

### Electrophysiology

Activity of neuronal ensembles was recorded from PD (n = 7), CD (n = 4) and LC (n = 4) rats using the methods described by Rema and Ebner [41]. Rats were anesthetized with urethane (1.5 gm/kg body weight, I.P). Multi-neuronal activity recorded from L2/3 and L4 of barrel columns from left hemisphere, using a carbon-fiber microelectrode (0.8-1.0MΩ) was amplified (2 K), bandpass filtered (0.7-7 kHz), sampled at 20 kHz (Neurolog, Digitimer) and acquired using Spike-2 software (CED). L2/3 and L4 were considered to extend from 150-450 µm and 450-800 µm below pial surface respectively [42]. Prior to recording, all whiskers on the right side of the face were trimmed to a length of 10 mm beyond the fur. From each recording site 2 min of spontaneous activity was recorded followed by evoked responses to stimulation of the principal whisker (shortest latency, largest response magnitude) and 3-4 surround whiskers. Each whisker was stimulated with 50 stimuli (3 ms duration, 0.5 ms rise time, 500 µm deflection: rostral direction), at 3 s interstimulus interval, using a piezoelectric wafer actuated by a digital stimulator (DS8000, WPI). On completion of a recording session, the electrode position was marked with an electrolytic lesion and localized in tangential sections of the recorded hemisphere reacted for cytochrome oxidase enzyme [43].

### Analysis of electrophysiology data

Stimulus evoked multi-neuronal spike activity (~4-5 neurons) was separated from baseline activity using Spike-2 software. The mean spontaneous activity of neurons in each layer was determined (spikes/sec) from averaging the 2 min spontaneous activity recorded from all the sites for that layer. Cumulative spontaneous activity was calculated for 1 min and the mean spike rate/ms was subtracted from each 1 ms peristimulus time bin. Population peristimulus-time histogram for each whisker was constructed from 1 ms bins from action potentials generated between 10 ms pre-stimulus to 100 ms post-stimulus. Response magnitude for each layer was calculated from the total number of spikes evoked for 50 stimuli, in the 4-100 ms post-stimulus time period, averaged for all the sites that were recorded from that layer. Response onset was determined as the latency (onset latency) at which at least three spikes occurred within 4-20 ms post-stimulus [44] and the peak response latency was the bin that had the maximum number of spikes between 4 and 20 ms post-stimulus.

### Immunohistochemical detection and measurement of NMDAR1 and GAD65

Levels of N-methyl-D-aspartate receptor subunit1 (NMDAR1) and glutamic acid decarboxylase 65 (GAD65) protein in L2/3 and L4 of the barrel cortex were examined by immunohistochemistry from PD (n = 8) and control (n = 5) rats. Electrophysiological recording was done on the brains of six of the PD rats and two of the control rats. The procedure for immunohistochemistry was as previously described [45] with slight modifications. Coronal sections were treated with antibodies to either NMDAR1 or GAD65 (BD Pharmingen) at 4°C for 36 hrs. Incubation was for 2 hrs with biotinylated secondary antibody (Vector Laboratories, Burlingame, CA), followed by ABC reagent (Vector Laboratories) for 2 hrs at room temperature. Chromogenic detection of NMDAR1 and GAD65 was carried out using diaminobenzidine and H_2_O_2_ as substrates. One series of sections was stained with cresyl violet for identifying cortical layers.

Images were acquired on a Nikon bright field microscope (Nikon Eclipse E800) using a digital camera (Optronics Microfire). From the barrel cortex of each rat, four sections (sections separated by 600 µm) were used for density measurements. Microscope setting, illumination and exposure times were identical for acquiring images from experimental and control sections. Digitized images were converted to eight-bit grayscale. Intensity of the staining was determined by measuring mean pixel density from images using NIH ImageJ software (National Institutes of Health, Bethesda, MD). Density measurements were made from L2/3 and L4 of the barrel cortex of each image. Background staining from the density measurements of each image was eliminated by subtracting the mean pixel density of regions lacking immunolabeling within that image. The results are presented as average relative intensity values (mean ± SEM) for L2/3 and L4 from the PD and control rats.

### Statistical Analysis

All data collection and analyses parameters were kept identical for control and experimental groups of animals. Data analyzed using Neuroexplorer and Microsoft Excel softwares are presented, unless otherwise indicated, as mean ± SEM. Box plots were constructed using SigmaPlot. Statistical analyses for all experiments (behavior, electrophysiology and immunohistochemistry) were performed with SigmaStat statistical tool-pack. The data from PD, CD and LC groups were tested for normal distribution and homogeneity of variance and the differences between the three groups were analyzed by one-way analysis of variance (ANOVA) and post-hoc test of Tukey. A P-value ≤ 0.05 was considered statistically significant with * = p ≤0.05, ** = p ≤ 0.01, *** = p ≤ 0.001.

## Results

Our goal was to characterize the nature of cellular deficits in the somatosensory cortex associated with behavioral impairments in adult rats following chronic protein deficiency. We exposed rats to protein deficiency from gestation up to adulthood. Using the ‘Gap-crossing’ task we quantified whisker-mediated somatosensory cortex-dependent behavior of protein-deficient and control rats. To understand the nature of deficits in sensory neurotransmission we recorded extracellular multiunit activity from L4 and L2/3 from barrel cortex of PD and control rats. We also examined the levels of GAD65 and NMDAR1 in barrel cortex of PD and control rats.

### Effect of chronic protein deficiency on body length and weight

**Figure 1:**
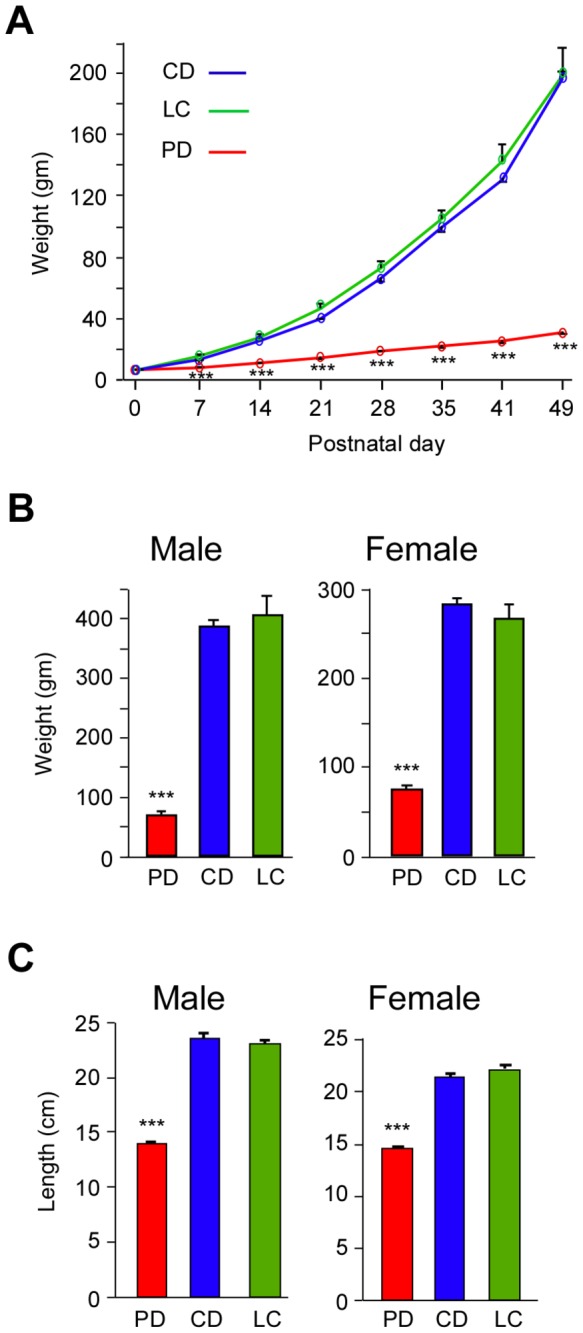
Weight and body-length of PD and control rats. *A*, Mean body weight of PD and control (‘CD’ and ‘LC’) pups at different ages. There is no significant difference in the birth weight of PD and control pups on postnatal day 0. The difference in body weight between PD and control pups were highly significant at all other ages. *B*, Mean weight and ***C***, mean body-length of 3-4 month old male and female PD rats and age matched control (‘CD’ and ‘LC’) rats that were used for gap-crossing experiment (note difference in maximum value on Y axis in ‘***B***’). Control males weighed more than control females, whereas the PD males weighed less than PD females. Difference between CD and LC controls were not significant. *** p ≤ 0.001. PD: protein-deficient diet fed rats, CD: control diet fed rats; LC: laboratory chow fed rats.

In order to determine the effects of protein-deficient diet on the growth of PD rats we measured their body weight and compared it to the CD and LC control rats. The weight of PD, CD and LC rats at selected ages up to seven weeks are shown in Figure 1A. On the day of birth there was no significant difference in the weight of PD pups compared to that of CD and LC pups (PD: 5.7 ± 0.3 gm, n =12; CD: 5.9 ± 0.1 gm, n = 25; LC: 6.1 ± 0.1 gm, n = 27; one-way ANOVA F(2,61) = 2.123, p = 0.128). On P1 the PD pups showed very small but significant decrease in weight compared to CD controls and LC controls. We found that this reduction in body weight of PD pups continued to persist up to adulthood (Figure 1A). The adult PD rats weighed significantly less than age matched controls (PD male: 68.5 ± 3.7 gm; CD male: 385.4 ± 12.4 gm; LC male: 404 .7 ± 33.1 gm; one-way ANOVA (F2,21) = 176.933, p < 0.001; PD female: 74.6 ± 4.3 gm; CD female: 281.2 ± 7.3 gm; LC female: 266 gm; one-way ANOVA (F2,17) = 157.536, p < 0.001) (Figure 1B).

We also measured the body-length (tip of the nose to base of the tail) of 3 month old rats, because the performance of the GC behavior is influenced by the body-length. The body-length of PD rats was significantly reduced compared to controls (PD male: 13.9 ± 0.25 cm; CD male: 23.6 ± 4 cm; LC male: 23.1 ± 4 cm; one-way ANOVA (F2,21) = 306.051, p < 0.001; PD female: 14.5 ± 0.1 cm; CD female: 21.3 ± 0.4 cm; LC female: 21.9 ± 0.4 cm; one-way ANOVA (F2,17) = 241.746, p < 0.001) (Figure 1C).

### Effect of chronic protein deficiency on gap-crossing behavior

**Figure 2:**
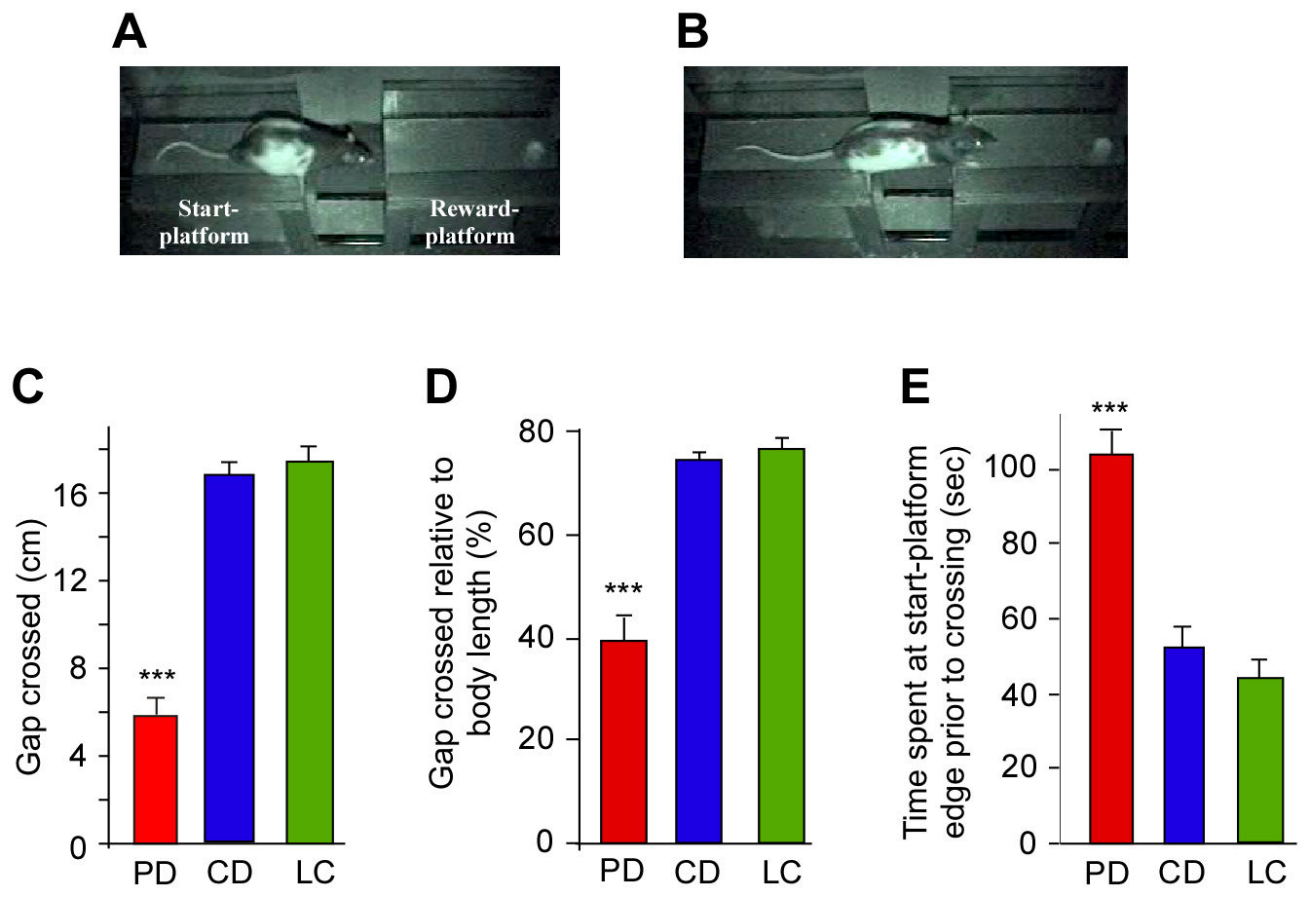
Performance on the GC task by PD and control rats. *A*, *B*, Video frames of a control rat contacting the reward-platform with its whiskers (*A*) and crossing the gap (*B*). *C*, Width of gap crossed by PD, CD and LC rats. *D*, Gap crossed by PD, CD and LC rats normalized to the body-length to control for differences in size. *E*, Time spent by PD, CD and LC rats at the start-platform prior to performing the final successful jump. The differences between CD and LC controls were not significant. *** p ≤ 0.001. PD: protein-deficient diet fed rats, CD: control diet fed rats; LC: laboratory chow fed rats.

Rats were tested on the performance on the GC task to test somatosensory whisker-mediated behavior. The GC task has been previously shown to be dependent on the somatosensory cortex. Since the task is performed in the dark, detection of the reward platform by the rat is mediated by contacting the edge of the reward-platform with their whiskers prior to jumping across the gap (Figure 2 A,B). The gap crossed by the PD rats was considerably smaller than that crossed by controls (PD: 5.6 ± 1.1 cm, n =5; CD: 16.6 ± 0.6, n = 5; LC: 17.4 ± 0.7, n = 5; one-way ANOVA F(2,12) = 94.521 p < 0.001) (Figure 2C). It is likely that the PD rats were crossing narrower gaps because they were much smaller in length and not because of somatosensory deficits. Hence, the width of the gap jumped by each rat was normalized to its body-length. After normalization of the GC performance of each animal to account for differences in body length, we found that the average width of the gap crossed by PD rats was 38.6 ± 7.8% of their body length, while width of the gap crossed by CD and LC controls was 74.6 ± 1.1% and 76.9 ± 2.3% of their body-length respectively (one-way ANOVA F(2,12) = 42.882, p < 0.001) (Figure 2D). In addition, the PD rats spent almost twice as long before initiating a response at the edge of the start platform prior to crossing the largest gap (PD:104.2 ± 5.7 s; CD = 52 ± 5.4 s; LC: 43.6 ± 5.3 s; one-way ANOVA F(2,12) = 36.173, p < 0.001) (Figure 2E). The difference in time between the two control groups, CD and LC, was not significant (one-way ANOVA p < 0.3).

### Effect of chronic protein-deficiency on neuronal activity in the whisker barrel cortex

**Figure 3:**
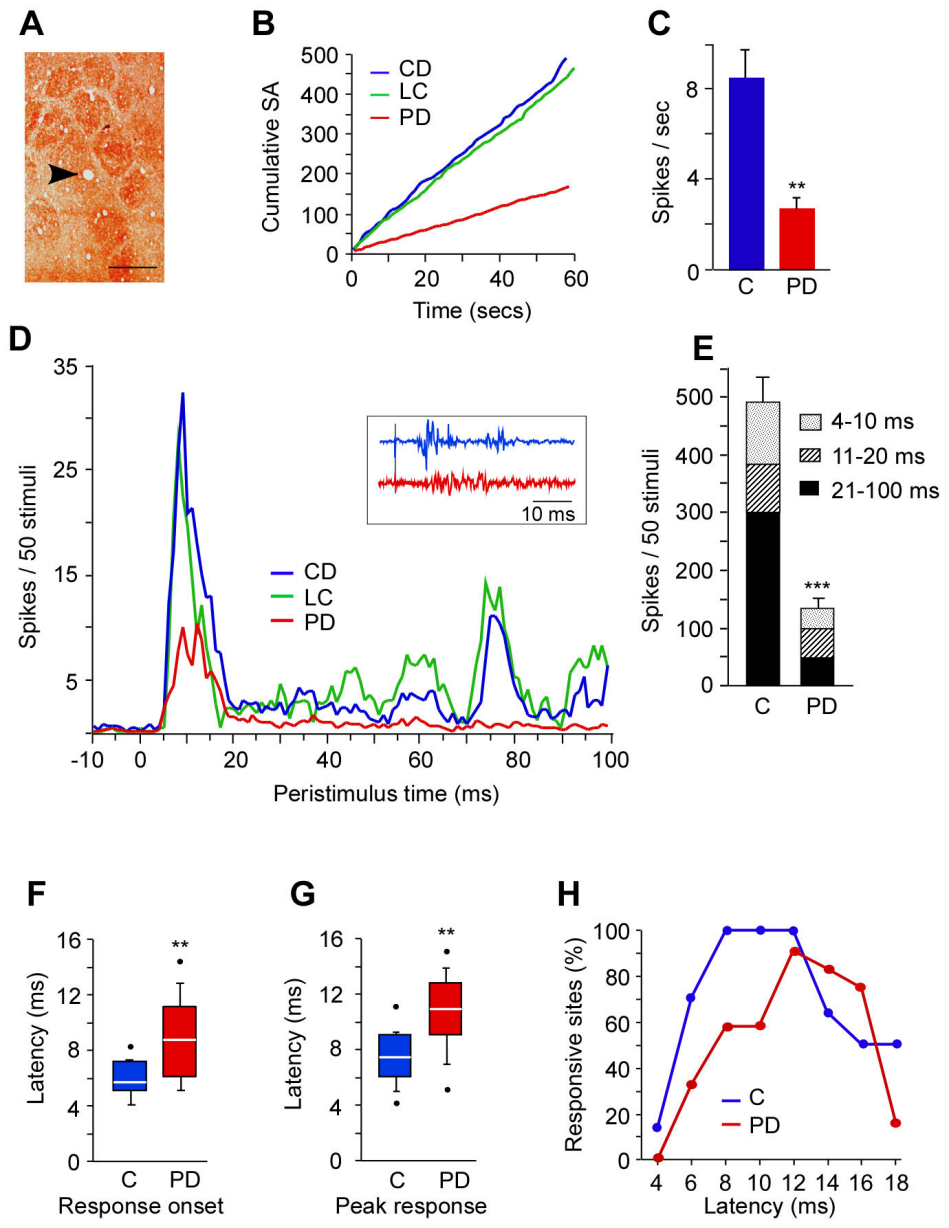
Activity of L4 barrel neurons in PD and control animals. *A*, Image of a tangential section through the barrel cortex of PD rat, arrowhead shows the recorded site in the D3 barrel marked with a lesion, scale bar = 500 µm. *B*, Cumulative multiunit spontaneous activity (SA; spikes/s) over a 1 minute epoch from PD, CD and LC rats. *C*, Spontaneous discharge rate shown as average number of spikes generated per second (± SEM) of L4 neurons from PD and control (‘C’: CD and LC combined) rats. *D*, Population peristimulus-time histograms of L4 multiunits from PD, LC and CD rats. Stimulus onset is at 0. Note the small initial response and severely depressed long latency response in PD rats (red line). Inset: Representative waveforms recorded from L4 neurons from a site in PD and control rat barrel cortex. Stimulus onset indicated by the grey vertical line across both waveforms. *E*, Total response magnitude, showing three pattern-coded latency epochs, is greatly reduced in PD rats compared to controls (‘C’: mean value ± SEM from LC and CD combined). *F*, Response onset latency, ***G***, peak response latency, and *H*, number of responsive sites (%) at different latencies emphasize the delay in response of L4 neurons in PD rats compared to controls (‘C’: mean value ± SEM from LC and CD combined). For details see ‘Methods’ and ‘Results’. ** p ≤ 0.01, *** p ≤ 0.001. PD: protein-deficient diet fed rats, CD: control diet fed rats; LC: laboratory chow fed rats. Note: As there is no statistical difference between the data from CD and LC groups, the data from both groups were combined as a single control group in ***C***, ***E***, ***F***, ***G*** and ***H***.

Performance of the “GC task” is dependent on intact barrel cortex. In addition whisker-dependent texture discrimination also requires intact barrel cortex. Therefore, the deficits in the performance of the “GC task” by PD rats indicated dysfunction of barrel cortex. To show the correlated effects of chronic protein deficiency on sensory processing we recorded multiunit activity from L4 and L2/3 of barrel columns (Figure 3A) in PD rats.

### Neuronal activity in thalamocortical input layer (L4) neurons

What is the effect of chronic exposure of protein deficiency on cortical neurons that are driven by direct thalamic inputs? Does protein deficiency alter spontaneous discharges of L4 neurons and their responsiveness to sensory stimuli? Examination of spontaneous activity of L4 neurons in PD animals showed a significant reduction in resting state activity. The cumulative spontaneous activity for 60 secs shown in Figure 3B reflects a 68% reduction in the mean spontaneous rate of L4 neurons in PD rats (2.8 ± 0.5 Hz, n = 12 sites) compared with controls (CD: 9.3 ± 2.6 Hz, n = 6; LC: 7.6 ± 1.1 Hz, n = 8, one-way ANOVA F(2,23) = 6.645, p < 0.01) (Figure 3C). The mean value is from a large sample of PD and control recordings, but the difference could be biased by fewer cells contributing to the spontaneous activity recorded in the multiunit recording. But that factor alone is unlikely to account for the entire difference. Indeed, a small sample of single cell waveforms suggested that neurons in control cases were more active than those from PD animals.

Responses of neurons within the layer 4 barrel to stimulation of the principal whisker were recorded from PD rats (12 sites) and compared to the stimulus evoked responses of CD (6 sites) and LC (8 sites) controls. The population peristimulus time histograms (Figure 3D) show a striking reduction in both the short latency and longer latency components of the response of L4 neurons in PD animals. Comparison of the total number of spikes evoked in 100 ms following stimulus onset showed that the response magnitude of L4 neurons in PD was ≤ 25% of controls (PD: 136.7 ± 17.9 spikes; CD: 540.2 ± 65.43 spikes; LC: 622 ± 40.49 spikes, one-way ANOVA F(2,23) = 61.54, p<0.001). To better understand the effect of PD on thalamic and intracortical inputs within L4, the responses in three latency epochs, 4-10 ms, 11-20 ms and 21-100 ms were examined (Figure 3E). The activity in the 4-10 ms reflects predominantly thalamic (VPM) input activity, while the activity in the 11-20 ms epoch comprises of both thalamic and intracortical inputs and the activity in the >21 ms period is dominated by intracortical inputs. In the 4-10 ms epoch the response magnitude in PD rats was reduced by ~65% compared with controls (PD: 34.8 ± 10.9 spikes; CD: 108.7 ± 10.9 spikes; LC: 103 ± 10.9 spikes; one-way ANOVA F(2,23) = 14.302, p < 0.001). Whereas, the reduction of response in the 11-20 ms epoch in PD rats was not significant as determined by one-way ANOVA F(2,23) = 1.311, p = 0.285 (PD = 90 spikes vs CD = 84.6 spikes and LC = 74.5 spikes). Most severe depression in response magnitude was seen in the 21-100 ms latency epoch at < 75% (one-way ANOVA F(2,23) = 66.570, p < 0.001).

We found that chronic protein-deficiency affected the temporal dynamics of sensory information processing in L4. The response onset in PD rats was delayed approximately by 3 ms (PD = 8.6 ± 0.8ms; CD = 5.83 ± 0.3; LC = 5.75 ± 0.5; one-way ANOVA F(2,23) = 6.935, p = 0.004) (Figure 3F). The latency at which the L4 neurons responded maximally (peak response latency) was also delayed in PD animals by ~3 ms (PD: 10.6 ± 0.8 ms; CD: 7.1 ± 0.4 ms; LC: 7.4 ± 0.5 ms; one-way ANOVA F(2,23) = 6.212, p = 0.007) (Figure 3G). Figure 3H shows the responsiveness of all sites in L4 of PD and control rats during 4-20 ms following stimulation of principal whisker. In controls at 4 ms poststimulus 14% of all sites produce a response and by 7 ms 93% sites had responded. By 8 ms poststimulus all the sites (100%) responded to the stimulation and they maintained their responsiveness for 5 ms i.e. until 13ms post-stimulus. However in PD rats not all recorded sites responded to stimuli. At 4 ms post-stimulus none of the sites produced a response. Response was seen in 17% of the sites at 5ms poststimulus which increased to 33% sites by 7 ms. At 12 ms the number of responsive sites in PD rats was maximum at 92%. The responsiveness of these sites was not maintained beyond 12 ms.

### Neuronal activity in L2/3

**Figure 4:**
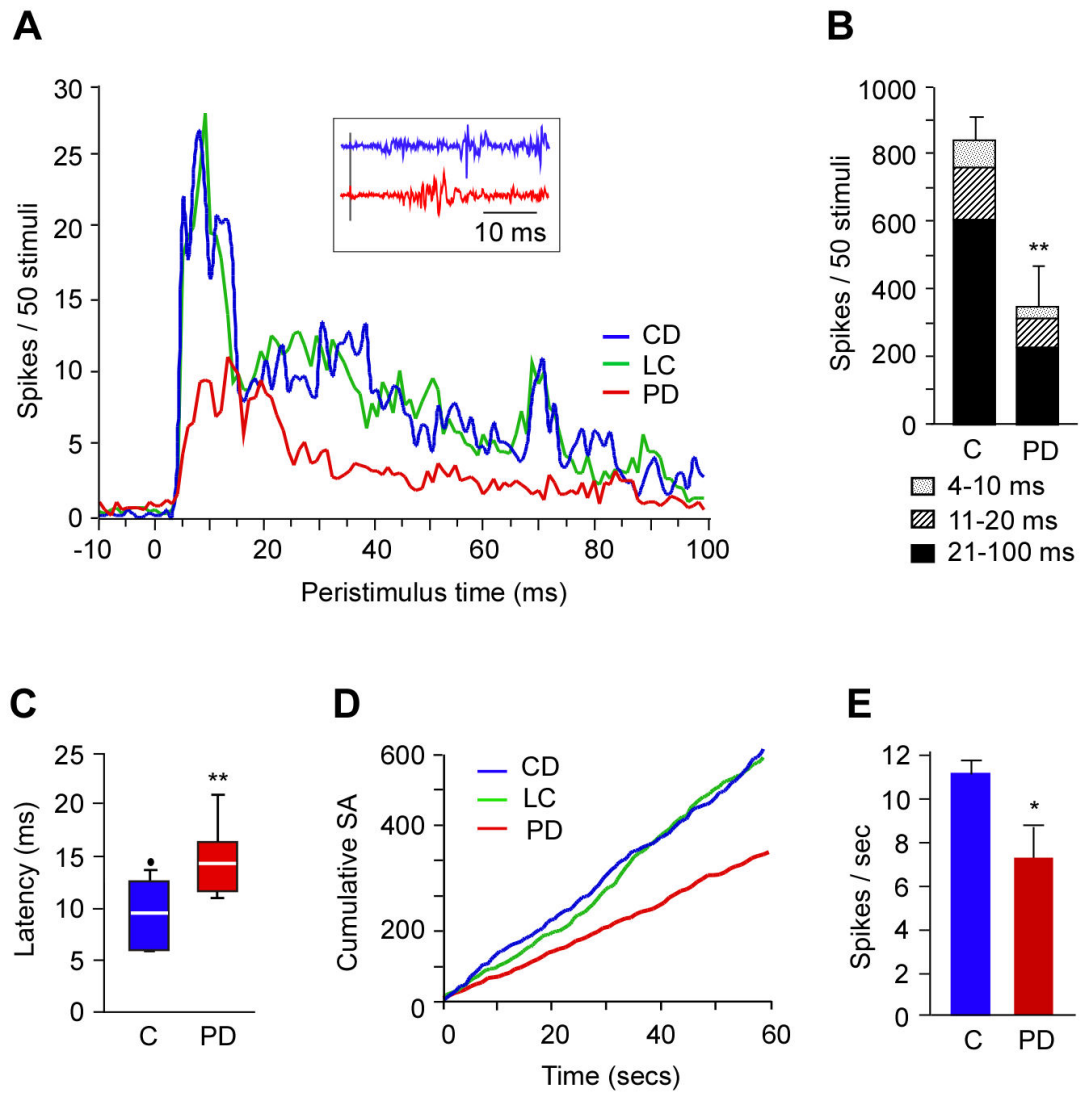
Activity of L2/3 neurons in the barrel cortex of PD and control rats. *A*, Population peristimulus-time histogram showing averaged activity of all recorded sites in PD, CD and LC rats. Inset in ‘***A***’: Representative waveforms recorded from L2/3 neurons from a site in PD and control rat barrel cortex. Stimulus onset indicated by the grey vertical line across both waveforms. *B*, Total response magnitude, depicting responses in three pattern-coded latency epochs, shows reduction in responses of L2/3 neurons in PD compared to control (‘C’: mean value ± SEM from CD and LC combined) rats. *C*, Response onset latency of L2/3 neurons of PD rats is longer compared with controls. *D*, Cumulative spontaneous activity, (‘SA’: spikes/s) and *E*, the mean spontaneous rate of L2/3 neurons of PD rats are lower than that of controls (‘C’: mean value ± SEM from CD and LC combined). For details see ‘Methods’ and ‘Results’. * p ≤ 0.05, ** p ≤ 0.01. PD: protein-deficient diet fed rats, CD: control diet fed rats; LC: laboratory chow fed rats. Note: As there is no statistical difference between the data from CD and LC groups the data from both groups were combined as a single control (‘C’) group in B, C, and ***E***.

The somatosensory information from L4 gets relayed to neurons in L2/3 for further intracortical processing. To what extent does chronic protein deficiency affect the L2/3 neurons? Neurons in L2/3 of PD animals responded with low magnitude to stimulation of the principal whisker (Figure 4A). The mean response magnitude was significantly lower than the controls (PD: 351.3 ± 123.7 spikes, CD: 796.5 ± 114.85 spikes; LC: 894 ± 81.847 spikes; one-way ANOVA F(2,16) = 7.031, p = 0.006) reflecting the reduction in responses in the three latency epochs in PD rats. While reduction of responses in the 4-10 ms and the 20-100 ms epochs were significant (4-10 ms: PD = 31 ± 11.9 spikes, CD = 75 ± 11.76 spikes; LC = 85.83 spikes, one-way ANOVA F(2,16) = 7.038, p = 0.006; 21-100 ms: PD = 230.7 ± 108 spikes, CD = 566 ± 105 spikes, LC = 629 ± 82.5 spikes, one-way ANOVA F(2,16) = 4.718, p = 0.025), there was no significant reduction in response magnitude in the 11-20 ms epoch (PD = 87.9 ± 26 spikes, CD = 126.8 ± 22.9 spikes; LC = 154.2 ± 24 .71 spikes, one-way ANOVA F(2,16) = 1.856, p = 0.188) (Figure 4B). We also found that the L2/3 neurons in PD animals were slower in responding to sensory stimuli. The peak response latency for PD animals was longer by ~6 ms than controls (PD: 15 ± 1.8 ms, CD: 9 ± 0.9 ms, LC: 8.8 ± 0.8 ms, one-way ANOVA F(2,11) = 8.13, p < 0.01) (Figure 4C). This indicated delay in the transmission of sensory information to the supragranular layers in PD rats. Neurons in L2/3 of PD rats had low spontaneous activity compared with controls (Figure 4D). However, the ~35% reduction in mean spontaneous activity in L2/3 of PD rats (PD = 7.2 ± 1.4 Hz vs CD = 12.4 ± 1.2 Hz & LC = 10.5 ± 1.4 Hz; one-way ANOVA F(2,13) = 5.424, p < 0.05) (Figure 4E) was relatively less than the ~63% reduction seen in the L4 neurons.

### Effect of chronic protein deficiency on responses of surround whiskers

**Figure 5:**
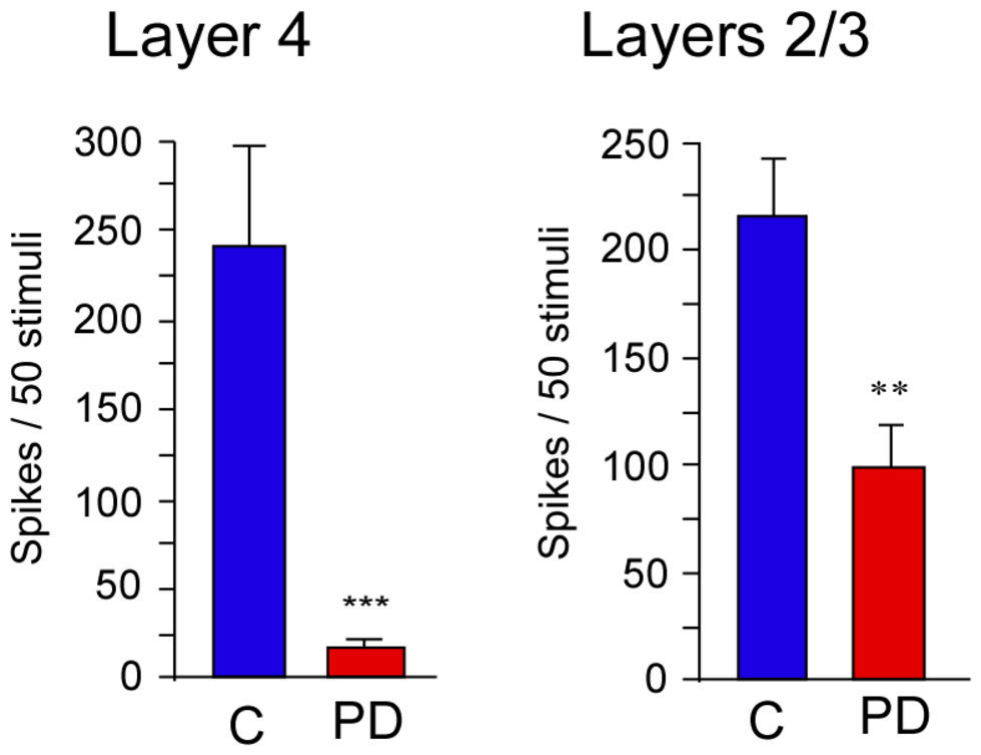
Response magnitudes of neurons in L2/3 and 4 of barrel cortex to stimulation of surround whiskers in PD and control (‘C’) rats. Note the highly significant reduction in the response of L4 neurons in PD rats to stimulation of surround whiskers. ** p ≤ 0.01, *** p ≤ 0.001. PD: protein-deficient diet fed rats. Note: As there is no statistical difference between the data from control diet fed rats and laboratory chow fed rats the data from both groups were combined as a single control ‘C’ group.

We estimated the response magnitude of neurons in L2/3 and L4 to stimulation of the surround whiskers, i.e. those adjacent to the principal whisker, to determine the intracortical transmission of somatosensory information and the surround receptive field (Figure 5). The average response magnitude of neuronal ensembles from each site in L4 of PD rats to surround whisker stimulation was extremely small (16.8 ± 4 spikes) compared to controls (CD: 236.3 ± 87 spikes; LC: 205.7 ± 58.5 spikes one-way ANOVA (F2,22) = 6.165, p = 0.007). Similar reduction in responses to stimulation of surround whiskers was seen in L2/3 neurons (PD = 98.3 ± 19.4 spikes; CD = 220.4 ± 18.9 spikes; LC = 212.8 ± 20 spikes, one-way ANOVA F(2,43) = 12.049, p < 0.001). There was no significant difference between the CD and LC controls in L2/3 and L4. Although L2/3 neurons in PD rats had 54% decrease response magnitude to stimulation of surround whiskers, the acute reduction of 93% in L4 neurons shows that chronic protein deficiency affected L4 neurons with more severity.

### Chronic protein deficiency does not reduce the amount of NMDAR1 subunit protein in barrel cortex of adult rats

**Figure 6:**
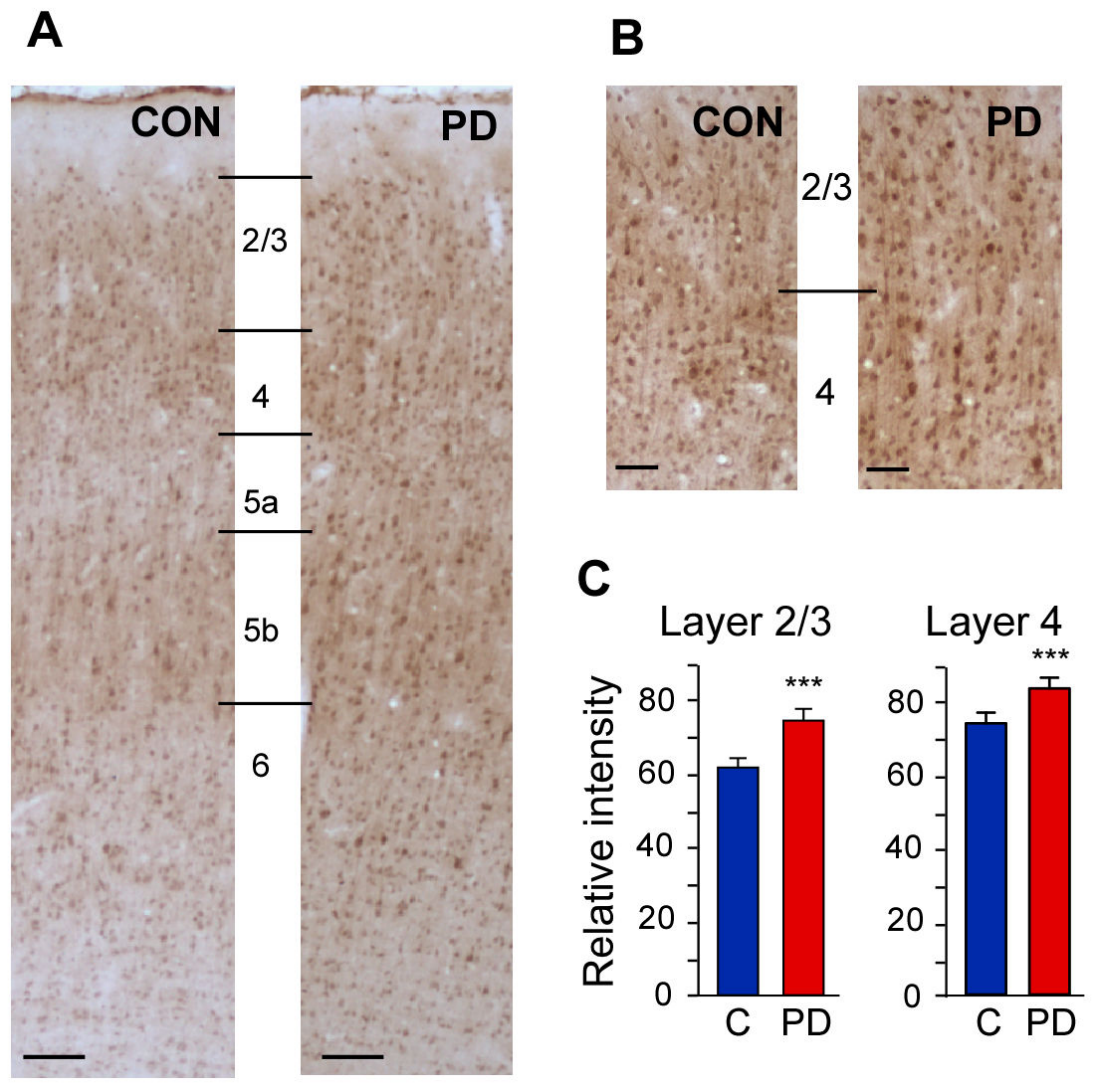
Photomicrographs showing pattern of NMDAR1 immunoreactivity in PD and control (CON) rats, *A*, through all layers of the barrel cortex, and *B*, in L2/3 and L4 at higher magnification. Laminar boundaries for cortical layers 1 to 6 are indicated by horizontal lines. Scale bar in A, 100 µm; B, 50 µm. *C*, Relative intensity of NMDAR1 staining in L2/3 and L4 in PD rats is slightly, but significantly, higher than control (‘C’) rats. Note: As there is no statistical difference between the data from control diet fed rats and laboratory chow fed rats the data from both groups were combined as a single control ‘C’ group. *** p ≤ 0.001. PD: protein-deficient diet fed rats.

The reduction in response magnitude of neurons from PD animals could be due to the dysfunction in excitatory neurotransmission. Since the most severe decrease was in the long latency NMDA receptor-dependent responses we examined the amount of NMDAR1 protein, the obligatory subunit of NMDA receptor, in the barrel cortex of PD rats (Figure 6). Our results show that exposure to protein-deficient diet throughout lifespan does not cause reduction in the level of NMDAR1 subunit in layers 2/3 and 4 of somatosensory whisker barrel cortex of rats (Figure 6 A,B). Rather, the quantification of NMDAR1 staining revealed a small but significant increase in the NMDAR1 expression in cortical layers 2/3 and 4 of barrel column of PD rats compared to control rats. The relative intensity of NMDAR1 in layers 2/3 was 74.6 ± 2.7 for PD rats, whereas it was 61.5 ± 2.1 and 62.2 ± 0.6 for CD and LC rats respectively (one-way ANOVA F(2,340) = 79.67, p < 0.001) (Figure 6C), representing a 20% increase in NMDAR1 protein in the PD rats. In layer 4 there was a 12% increase in the relative intensity of NMDAR1 in PD rats (82.5 ± 3.2) compared with control rats (CD: 74.86 ± 1.12; LC: 71.55 ± 0.74, one-way ANOVA F(2,201) = 103.502, p < 0.001) (Figure 6C). There was no significant difference in the levels of NMDAR1 between the two controls.

### Chronic exposure to protein deficiency increases GAD65 protein in the barrel cortex of adult rats

**Figure 7:**
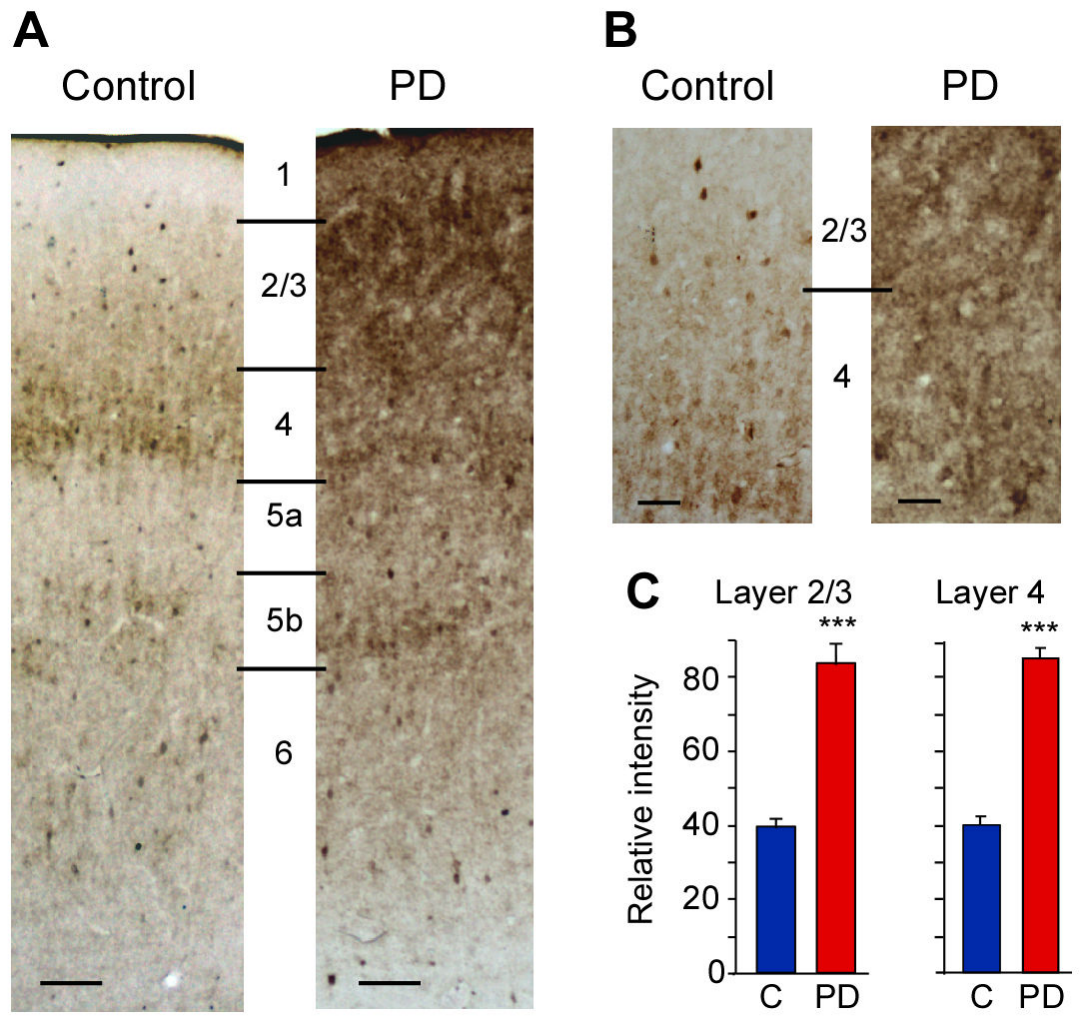
Photomicrographs showing pattern of GAD65 immunoreactivity in PD and control (CON) rats, *A*, through all layers of the barrel cortex, and *B*, in L2/3 and L4 at higher magnification. Laminar boundaries for cortical layers 1 to 6 are indicated by horizontal lines. Scale bar in A, 100 µm; B, 50 µm. *C*, Relative intensity of GAD65 staining in L2/3 and L4 in PD rats is highly significant than control (‘C’) rats. Note: As there is no statistical difference between the data from control diet fed rats and laboratory chow fed rats the data from both groups were combined as a single control ‘C’ group. PD: protein-deficient diet fed rats. *** p ≤ 0.001.

The reduction in response magnitude of neurons in the barrel cortex of PD rats to sensory stimulation could reflect enhanced inhibition. Hence, we examined the level of GAD65 in PD rats to provide an insight into the status of inhibition following chronic protein deficiency. GAD65 is present predominantly at the presynaptic terminals and is involved in the synthesis and release of GABA at synapses. The high level of GAD65 in L2/3 and L4 in the coronal sections of barrel cortex in PD animals was striking as seen in Figure 7A,B. The amount of GAD65 in L2/3 as estimated by relative intensity measurement was 83.9 ± 5.6 in PD rats vs 40.7 ± 0.9 and 38.8 ± 1.1 in CD and LC rats respectively (one-way ANOVA F(2,265) = 203.65, p < 0.001) (Figure 7C). The relative intensity of GAD65 staining in L4 (PD = 85 ± 2.6 vs CD = 41.2 ± 0.9, LC = 39.9 ± 1.0; one-way ANOVA F(2,299) = 644.39, p < 0.001) (Figure 7C) was very similar to that seen in layers 2/3. In L2/3 and L4 there was no significant difference in the levels of GAD65 between the CD and LC controls. Both L2/3 and L4 showed 112% increase in GAD65 levels in PD rats compared with control rats.

## Discussion

In these experiments we used behavior and extracellular multineuron recording to investigate the effects of long-term exposure to protein-deficient diet from gestation to adulthood on somatosensory functions in adult rats. We found dysfunctions in the somatosensory cortex-dependent whisker-sensitive GC behavior in PD rats. The somatosensory whisker-barrel cortex neurons of PD rats showed (i) severe reduction in spontaneous and evoked activity in the main input layer, L4; (ii) low spontaneous and evoked activity of L2/3 neurons; (iii) temporal delay in responding to sensory stimuli as seen by increase in onset and peak response latencies; (iv) reduction of responsive sites and inability in maintenance of excitatory drive in L4 neurons and (v) diminutive response to surround whisker stimulation. We also found a very high increase in the level of GAD65 protein. These results demonstrate, for the first time, that chronic protein deficiency interferes with processing of tactile information by the somatosensory cortical neurons. Such neurophysiological deficits in the brain could hence form key underlying factors in causing cognitive and behavioral dysfunctions.

Resnick et al. [46], have shown that very low protein diet (6% casein) consumed by dams during pregnancy causes low birth weight pups mimicking infantile marasmus seen in humans, while the pups of dams that consumed diet with 8% casein had normal birth weight. Other studies from this group have shown that despite normal birth weight, the 8% casein-fed pups displayed significant elevations in levels of seretonin, norepinephrine and their precursors [46]. In our experiments, since we found that the pups born to 7% casein-diet fed dams had normal birth weights, we assumed that the detrimental effect of protein-deficiency is not as severe as the 6% casein-fed pups seen by Resnick’s group [46]. Although the PD pups in our study do not exhibit symptoms of infantile marasmus it is possible that changes in the chemical milieu of brain might occur as has been shown for the 8% casein diet fed pups [46]. Such alteration in brain molecules could in turn affect metabolic pathways in the brains of protein deficient rats. For example, reduction in glutamate uptake [47], increase in GABA uptake [48] is seen in cerebral cortex and hippocampus of rats following chronic exposure to 7% casein diet. While there is no direct evidence that such molecular changes can be linked to alterations in brain structure and functions, mice with prenatal exposure to 7% casein-diet had structural alteration in the cerebellum and showed impairments in motor coordination and spatial memory [49,50]. Therefore, we cannot rule out the fact that similar alterations are possible in the barrel cortex of PD pups in this study.

Even though the weight of PD pups in our study were comparable to those of controls on P0 they became slightly but significantly smaller than the control pups on P1 and weighed ~30-40% less than controls by P7. Similar reduction in body weight by P5 has been reported by Resnick et al. [46] in 8% casein-fed pups. These authors attributed the reduction in weight to production of lower volume of milk by lactating dams who were consuming protein deficient diet as was shown by Mueller and Cox Jr [51]. It was found that the lactating dams who were ingesting low protein diet were producing milk with lower nitrogen content [52], lesser concentration of milk lactose and reduction in milk protein [53]. Further experiments are needed in determining the exact effect of quantity or quality of milk during lactation on neuronal function of protein deficient animals.

Quantifiable measures of behavioral and neurophysiological deficits are required to understand the effect of exposure to chronic protein deficiency on brain functions. We took advantage of the extensively studied somatosensory information processing in the rat whisker barrel cortex [54] and whisker-mediated, somatosensory cortex-dependent behavior assessed by the GC task [39,55-57]. Similar to stunting in children affected by protein deficiency [58], the PD rats were small [16,59]. The PD male rats were smaller than PD female rats indicating that the male rats were affected more by protein-deficiency. Hence, we normalized the performance of GC task of each animal to its body-length. Control rats crossed gaps that were 74.8 ± 2.9% body-length aided by contact of reward platform with their whiskers. In contrast, the PD rats did not cross gaps wider than 38.6 ± 7.8% body-length despite contacting the reward platform with forepaws and snout in addition to whiskers. They also spent longer time probing the reward platform prior to crossing. The poor behavioral performance of the PD rats could be due to motor deficits resulting from muscle weakness. However, we observed that the PD rats were able to support their body weights for longer time with their forelimbs on ‘wire hanging test’ compared to controls. In fact we observed that some of the PD rats pulled themselves up on to the wire and walked on it, while the control rats released their grip and landed in the home cage below (data not shown). In addition, the PD rats were more active than controls in ‘open field test’ (data not shown), indicating that the impaired GC behavior was not because of movement deficits.

Although, GC behavioral deficits suggested dysfunction of the neurons in the somatosensory cortex of PD rats in processing somatosensory information it was not clear whether the behavioral impairments were learning deficits. This prompted us to examine the physiological and molecular status of the somatosensory cortex. Our multiunit recordings of neuronal ensembles from the main input layer (L4) and the cortico-cortical processing layers (L2/3) of barrel cortex of PD rats revealed that there was severe reduction in spontaneous activity. This finding is in agreement with the results of another study which showed that the neurons in the neocortex and thalamus of rats that were chronically exposed to 8% casein diet had low levels of spontaneous discharges [60]. Importantly, in this study we found that responses of the neurons in layer 2/3 and 4 to stimulation of principal whisker were significantly lower. The reduction in evoked activity was more pronounced in L4 and affected both short latency thalamocortical and long latency intracortical components of sensory response. The delays in the response onset and peak response indicate slower transfer of sensory information from the thalamus to the cortex. While the decrease in the response in the long latency epoch in L4 suggested deficits in intracortical sensory information processing. It has been shown that surround receptive field of neurons in L4 is generated intracortically [61]. Hence, the reduced responses of L4 neurons to surround whisker stimulation as well as the low responses of L2/3 neurons indicated impairment in cortico-cortical transmission of sensory information.

The neurophysiological deficits that we observed in the present study could be due to the structural alterations in the barrel cortex of PD rats. Chronic exposure to low protein diet have been shown to cause reductions in the barrel area in mice [25]. Our preliminary results from measurement of barrel area show that compared to controls the area occupied by each barrel is smaller in PD rats (data not shown). In addition, it is likely that the deficits in whisker-mediated sensory transmission is also caused by reduction in the number of neurons, as shown in the barrel field of mice [25] and in the visual cortex of rats [62] exposed to chronic protein deficiency. Also reduction in dendritic length and spine density [59] and synapse number [63] following prolonged protein deficiency could have adverse effect on neurotransmission.

We found that in PD rats the maximum reduction in evoked responses was in the long-latency component. The long-latency components of evoked responses of neurons in L2/3 and L4 of barrel column to principal whisker stimulation, as well as the responses to stimulation of surround whiskers have been shown to be dependent on the activity of excitatory amino acid receptor, the NMDA receptor [64,65]. Interestingly, the PD rats had low spontaneous and evoked activity in L2/3 and L4 of barrel columns despite a small but significant increase of NMDAR1, which has been shown to be the obligatory subunit of functional NMDA receptor channel [66]. In support of our observation, increase in the level of NMDA receptor, as indicated by an increase in striatal NMDA receptor binding and reduction in levels of prepulse inhibition was seen in adult rats who had prenatal exposure to protein deficiency [32]. However, Schweigert et al. [48], showed that rats exposed to protein deficiency during gestation and early postnatal period showed lower sensitivity to NMDA receptor agonist quinolinic acid suggesting reduction in NMDA receptors or alteration in NMDA receptor channel properties.

The decrease in neuronal activity in the barrel cortex of PD rats could be a result of enhanced inhibition. Modulation of the levels of the inhibitory amino acid GABA at the synapses could influence excitability of the neurons. GAD65 is primarily localized in the axon terminals [67,68] and is implicated in the synthesis and release of GABA at the nerve terminals in response to afferent activity [69,70]. Prenatal exposure to protein deficiency has been shown to cause differential modulation of GABA and GAD in rats during development in hippocampus and cortex. Decreases in the expression of GAD and release of GABA were seen at P21, whereas on P60 there was increase in stimulated GABA release and GAD expression [71]. However, exposure to prenatal protein deficiency reduced the levels of mRNA for GABA A receptor subtypes in the medial septum, lateral septum and hippocampus with no change in the GABA B receptor [72,73]. In our study the rats that were exposed to chronic protein deficiency had substantial increase in GAD65 in L2/3 and L4 suggesting that there could be enhanced GABA mediated inhibition during processing of somatosensory information while the rats are performing the behavioral task. We do not rule out the possibility that chronic protein deficiency can affect the levels of GAD67. Following chronic protein deficiency, alteration in number of GAD67 positive neurons has been reported in dentate gyrus and CA1-3 subfields of hippocampus [74]. It is also possible that in the barrel cortex of PD pups in our study, there are modifications in the subcellular distribution of GAD65 at the axon terminals and changes in number of GABAergic neurons.

The deficits in somatosensory neurotransmission and somatosensory behavior observed in the present study could be a consequence of the alteration in brain structure during early development resulting from protein deficiency during gestation and lactation. Several studies in animal models have shown that exposure of the fetus to protein deficiency during early stages of development (gestation and lactation) has adverse effect on brain functions. Behavioral impairments in attention, executive functions, conduct problems, fine motor skills, externalizing behavior and depression, as well as low IQ in adolescents and young adults exposed to protein energy malnutrition during early childhood have been reported [75,76]. Hence, rehabilitative programs have targeted children but positive outcomes have been limited [77,78].

However, consumption of protein deficient diet in adult ages could also cause deleterious effect on brain structures. Adult rats exposed to protein deficient diet showed cell loss in dentate gyrus, CA3 and cerebellum [79]. In addition, the effect of chronic protein deficiency has been shown to be more detrimental than prenatal protein deficiency alone [20,21,74,80]. This entails that adverse effects of PD diet are not limited to early development period. As mentioned by Morgane et al [22] “the classic and indeed most prevalent form of malnutrition in humans is characterized by its chronic nature, the lack of adequate amounts of high-quality protein in the diet, the substitution of fats and carbohydrates in the diet in place of protein, and the continuation of the inadequate diet throughout the life of the individual.” Hence, for the present study we exposed rats to chronic protein deficiency to simulate protein malnutrition in human populations living in poverty-related socioeconomic conditions.

We are yet to determine how the low spontaneous activity and responsiveness of barrel cortex neurons to sensory stimuli in PD rat would affect experience-dependent plasticity [81]. However, a previous study has shown that diminished responses of barrel cortex neurons following cortical injury had negative impact on experience-dependent plastic changes [82]. In another study it was seen that neurons in the barrel cortex of adult rats who were exposed to alcohol during gestation exhibited severe reduction in spontaneous as well as stimulus evoked responses [41]. These animals also had severe deficits in experience-dependent plasticity [41]. Results from these studies suggests that similar deficits in experience-dependent plasticity mechanisms are likely to occur in the PD pups.

Our results indicate that the suppression of activity of cortical neurons following chronic exposure to protein deficiency could potentially impair learning, memory and cognitive functions. The cognitive impairments in children affected by protein deficiency [3] can lead to adverse socioeconomic outcomes in adulthood [83]. The effect of nutritional rehabilitation of protein malnourished children has been partially positive [77,78]. However, the effectiveness of nutritional rehabilitation of adults who have been exposed to chronic protein deficiency is uncertain. Nutritional rehabilitation of adult rats maintained on PD diets from gestation to adulthood was ineffective in improving the low spontaneous discharge rates of neurons from frontal cortex [84]. Understanding the mechanisms that cause these deficits may ultimately lead to effective rehabilitation for humans exposed to chronic protein-deficiency.
